# Hormone Treatment, Estrogen Receptor Polymorphisms and Mortality: A Prospective Cohort Study

**DOI:** 10.1371/journal.pone.0034112

**Published:** 2012-03-23

**Authors:** Joanne Ryan, Marianne Canonico, Laure Carcaillon, Isabelle Carrière, Jacqueline Scali, Jean-Francois Dartigues, Carole Dufouil, Karen Ritchie, Pierre-Yves Scarabin, Marie-Laure Ancelin

**Affiliations:** 1 Inserm U1061, Université Montpellier 1, Montpellier, France; 2 Inserm U1018, Université Paris Sud 11, UMRS 1018, F-94807, Villejuif, France; 3 Inserm U897, Université Bordeaux 2, Bordeaux, France; 4 Inserm U708, Paris, France; 5 Faculty of Medicine, Imperial College, London, United Kingdom; University of Washington, United States of America

## Abstract

**Background:**

The association between hormone treatment (HT) and mortality remains controversial. This study aimed to determine whether the risk of mortality associated with HT use varies depending on the specific characteristics of treatment and genetic variability in terms of the estrogen receptor.

**Methodology/Principal Findings:**

A prospective, population-based study of 5135 women aged 65 years and older who were recruited from three cities in France and followed over six years. Detailed information related to HT use was obtained and five estrogen receptor polymorphisms were genotyped. The total follow-up was 25,436 person-years and during this time 352 women died. Cancer (36.4%) and cardiovascular disease (19.3%) were the major causes of death. Cox proportional hazards models adjusted for age, education, centre, living situation, comorbidity, depression, physical and mental incapacities, indicated no significant association between HT and mortality, regardless of the type or duration of treatment, or the age at initiation. However, the association between HT and all-cause or cancer-related mortality varied across women, with significant interactions identified with three estrogen receptor polymorphisms (p-values = 0.004 to 0.03) in adjusted analyses. Women carrying the C allele of *ESR1 rs2234693* had a decreased risk of all-cause mortality with HT (HR: 0.42, 95% CI: 0.18–0.97), while in stark contrast, those homozygous for the T allele had a significantly increased risk of cancer-related mortality (HR: 3.18, 95% CI: 1.23–8.20). The findings were similar for *ESR1 rs9340799* and *ESR2 rs1271572*.

**Conclusions/Significance:**

The risk of mortality was not associated with HT duration, type or age at initiation. It was however not equal across all women, with some women appearing genetically more vulnerable to the effects of HT in terms of their estrogen receptor genotype. These findings, if confirmed in another independent study, may help explain the differential susceptibility of women to the beneficial or adverse effects of HT.

## Introduction

Hormone treatment (HT) remains the treatment of choice for alleviating menopause-related symptoms which affect up to 80% of women in Western countries [1] and improving their health-related quality of life [2]. Weighing up the risks to benefits associated with the use of HT however, remains an important yet complex issue. It is known to reduce the risk of osteoporosis and fractures [3], but increases the risk of venous thromboembolism [4] and breast cancer [3]. Whether or not HT can modify the risk of mortality remains controversial. Although the majority of observational studies indicate that HT is beneficial [5], [6], [7], [8], reducing coronary heart disease risk and cardiovascular-related deaths [9], [10], [11], this has not been supported by the predominantly non-significant findings from large randomized controlled trials (RCTs) [12], [13], [14], [15], [16]. The Women's Health Initiative Study actually found that conjugated equine estrogen (CEE) with medroxyprogesterone acetate (MPA) increased the risk of CV disease [15], although this was limited to the oldest group of women [17] and there was no significant increased risk of mortality. While the nature of a RCT enables the “healthy HT-user” bias to be minimized, the majority of RCTs have involved older postmenopausal women who were administered a specific form of oral synthetic treatment (CEE with or without MPA) over a relatively short duration [12], [14], [15]. Other forms of treatment given for different periods of time may have more beneficial effects on health-outcomes and survival. This could include natural estrogen-progestagen preparations composed of estradiol with or without progesterone [18], [19], transdermal rather than oral administration [4] and longer periods of treatment [6], [9], [20]. In addition, women participating in observational studies represent the “usual” clinical situation, where treatment is generally sought for the relief of menopausal-symptoms at a younger age. Increasing evidence suggests there is a “critical window period”, whereby initiating HT around the menopause but not later in life could specifically reduce coronary heart disease and overall mortality (see for review [21], [22]), although this has seldom been tested.

It is also possible that some women are genetically more susceptible to the effects of HT than others [23], [24], [25], which may help explain inconsistencies in the literature. The actions of estrogen occur in large part through intracellular activation of its two principal receptors (*ESR1* and *ESR2*), and allelic variants in the genes encoding these receptors could influence HT-mediated signal transduction [26]. The intracellular concentration of these receptors appears to be correlated to the cellular response to estrogens [27]. Polymorphisms of these receptors have been shown to modify the effect of HT on high-density-lipoprotein cholesterol [28] and breast cancer [23], but whether this can influence survival has not yet been examined.

This study investigated the association between HT and mortality by examining characteristics of HT, in particular the duration and type of treatment, and the period when treatment was first initiated. Estrogen receptor polymorphisms which could modify estrogen signalling were also examined to determine whether genetic variability may help explain different susceptibility to the effects of HT on mortality.

## Materials and Methods

### Ethics Statement

The study protocol was approved by the Ethical Committee of the University Hospital of Kremlin-Bicêtre (France) and written informed consent was obtained from all participants.

### Participants

The Three-City Study (3C) is a multi-centre prospective cohort study involving the French cities of Bordeaux, Dijon and Montpellier [29]. Eligible participants (aged at least 65 years and non-institutionalised) were recruited by random selection from the electoral rolls between 1999 and 2001. Three follow-up examinations were performed at 2, 4 and 6 years. Participants were administered standardised questionnaires by trained staff and underwent a number of clinical examinations.

Of the 5524 women initially recruited in the 3C Study who were not diagnosed with probable or possible dementia, eight participants were lost to follow-up, 219 women were missing data on HT use and 162 had incomplete data concerning the covariates. A sub-sample of 4463 women from the 5135 remaining had complete genotyping data. Compared to the analysed sample, participants not included in this analysis were more likely to be older, have a lower education level, physical incapacities, cognitive impairment, depressive symptoms (p-values<0.001) and comorbidity (p = 0.02). They were also more likely to have died during the follow-up period (p<0.001), but there was no significant difference in terms of HT use or estrogen receptor genotypes.

### Mortality

The exact date when participants died was obtained from death registries. The causes of death were defined based on medical records and interviews with the general practitioner and family members [30]. The principal cause of death was considered in this analysis and was coded according to the tenth revision of the International Classification of Diseases (ICD-10) as follows: cancer (ICD-10: C00-D48), circulatory disease which includes cardiovascular disease and stroke (ICD-10: I00-I99, R960), respiratory and infectious disease (ICD10: J00-J99), cachexia and diseases of the digestive tract (ICD-10: R64, K00-K93) and ill-defined causes (ICD-10: remaining R00-R99).

### Hormone treatment

Participants recorded current and past use of HT at inclusion and detailed information related to the treatment. Treatment use was validated by presentation of the prescription or the medication itself. Current users were classified according to the route of estrogen administration and the type of progestogen (progesterone or progestins). The duration of current HT and the timing of initiation of first treatment in relation to the menopause were also examined, with age at menopause being defined as one year without menses.

### Estrogen receptor polymorphisms

Fasting venous blood samples were taken from the participants at baseline. DNA was extracted from white blood cells (Puregene kit, Qiagen, France) and stored at −80°C. Genotyping was performed by Kbiosciences (Hoddesdon Herts, UK) using their competitive allele-specific PCR Single-Nucleotide Polymorphism (SNP) genotyping system (KASPar). The amplified PCR products were analysed by fluorescence scanning in a BMG labtech Pherastar scanner and the results were interpreted with their KlusterCaller 1.1 software. The error rate for the KASPar assay system is less than 0.3%.

The two most commonly studied *ESR1* polymorphisms were examined [24], [31], [32], [33], *rs2234693* and *rs9340799* (otherwise known as *Pvu*II and *Xba*I), which are located at position 397 and 351 of intron 1 respectively, and they appear to be functionally significant [34], [35]. Three *ESR2* polymorphisms with unknown functional consequences but showing potential causal associations with other hormone-related health outcomes [25], [36] were investigated: *rs1256049* (position 1082 of exon 5), *rs4986938* (position 1730 in the 3′-untranslated region of exon 8) and *rs1271572* in the promoter region.

### Covariates

Information was gathered at baseline on the participant's age, education level, living situation, consumption of alcohol and smoking status. Body mass index (BMI) was calculated as weight (kg) divided by the height squared (m^2^). The Centre for Epidemiology Studies Depression Scale (CES-D) [37] was used for the assessment of depressive symptoms (CES-D ≥16). Participants were classified as having physical activity limitations if they were unable to complete at least two tasks from either the Instrumental Activities of Daily Living (IADL) or the Activities of Daily Living (ADL) scales [38]. Cognitive function was assessed using the Mini-Mental State Examination (MMSE) [39] and participants scoring less than 26 were classified as cognitively impaired. Information on the health of the participants was obtained through detailed medical questionnaires, a complete inventory of drug use in the preceding month and from fasting blood samples. Participants were classified as having comorbidity if they suffered from one or more of the following chronic illnesses: vascular diseases (angina pectoris, myocardial infarction, stroke, cardiovascular surgery, bradycardia or palpitations), asthma, diabetes (fasting glucose≥7.0 mmol/l or reported treatment), hypercholesterolemia (total cholesterol ≥6.2 mmol/l), hypertension (resting blood pressure ≥160/95 mm Hg or treated) or thyroid problems.

### Statistical Analysis

Chi-squared tests compared the baseline characteristics of women according to their use of HT, as well as the characteristics of women who had died during the follow-up period and those who were still alive. Cox Proportional Hazard analysis modelled the risk of mortality during the follow-up period that was associated with HT use at baseline. The time scale used in the Cox Model was the age of participants at inclusion, which allowed us to account for the fact that the risk of mortality with age among the elderly is non-proportional, and Cox models with delayed entry were used [40]. Multivariate analysis also controlled for covariates which were significantly associated with mortality and which could potentially confound the relationship between mortality and HT use, including education, living situation, physical incapacities, cognitive impairment, depressive symptoms and comorbidity.

Chi-squared tests were used to compare the distribution of estrogen receptor genotypes with those predicted under the Hardy-Weinberg equilibrium and pair-wise linkage disequilibrium was estimated. Assuming a dominant model, comparing the dominant allele with the combined group of heterozygotes and homozygotes for the variant allele, a first-order interaction between the polymorphisms and current HT was examined by including a product term in the multivariate Cox models. This was based on our *a priori* hypothesis that estrogen receptor polymorphisms could moderate the effect of estrogen on mortality. When significant interactions were found, subsequent analysis was stratified by genotype to determine independent group effects. There was no indication of colinerality between the covariates in the adjusted models. SAS version 9.1 (SAS Institute, Inc., Cary, North Carolina) was used for all of the statistical analysis.

## Results

### Study population

The women in this study were aged from 65 to 100 years. Almost 14% were current users of HT and a slightly higher percentage (16.5%) reported past use (Table 1). Current HT users were younger and better educated compared with both past and never users and they were less likely to have physical activity limitations or comorbidity.

**Table 1:** Baseline characteristics of the 5135 female participants according to their use of hormone treatment.

Baseline Characteristic	Current HT (n = 714)	Past HT (n = 845)	Never (n = 3576)	Statistic (df)	p
	Mean (S.D.)			f	
Age (years)	70.3 (3.4)	73.2 (5.0)	75.2 (5.6)	280.9	<0.001
BMI (kg/m^2^)	24.3 (3.5)	25.2 (4.1)	25.5 (4.5)	23.9	<0.001
	**%**			**χ^2^**	
≥12 years of education	34.5	22.7	21.2	59.0 (1)	<0.001
Married or living with others	62.9	54.7	47.5	62.3 (1)	<0.001
High alcohol consumption (≥24 g per day)	5.0	4.2	4.0	1.4 (1)	0.51
Heavy smoker (≥10 pack years)	4.1	3.7	3.9	0.2 (1)	0.92
Physical activity limitations	4.0	9.2	12.2	44.7 (1)	<0.001
Comorbidity	36.6	45.7	49.0	37.3 (1)	<0.001
Cognitive impairment (MMSE <26)	5.9	7.1	6.8	1.0 (1)	0.61
Depressive symptoms (CES-D ≥16)	28.0	31.0	28.9	1.9 (1)	0.38
Centre				37.4 (2)	<0.001
Bordeaux	15.6	21.0	25.0		
Dijon	60.2	52.3	52.3		
Montpellier	24.2	26.8	22.7		

The total follow-up time for the study was 25,436 person-years, over a median 5.2 years (interquartile range 4.6–5.7 years) and during this period 352 women (6.9%) died. The majority of women died from causes related to cancer (36.4%) or circulatory disease (19.3%), including cardiovascular disease and stroke. A substantial number died from ill-defined causes (23.3%), as the result of multiple pathologies and frailty. Mortality was significantly associated with a number of the health related variables examined (Table 2), and women who died during follow-up were also older, less educated and more likely to live alone. In unadjusted chi-squared analysis (Table 3), women who reported the use of current treatment were less likely to die (2.2%), compared with past (6.2%) or never HT users (8.0%) (p<0.001). Current HT users were more likely to die of cancer but were less likely to die of cardiovascular disease, compared to past and never user. In the latter case only, this difference was significant.

**Table 2:** Baseline characteristics of the 5135 female participants according to their mortality status at follow-up.

Baseline Characteristic	Alive (n = 4783)	Died (n = 352)	Statistic (df)	p
	Mean (S.D.)		t	
Age (years)	73.9 (5.3)	78.6 (6.4)	−15.7	<0.001
BMI (kg/m^2^)	25.3 (4.3)	25.8 (4.9)	0.25	0.80
	**%**		**χ^2^**	
≥12 years of education	23.7	17.9	6.1 (1)	0.01
Married or living with others	51.4	42.1	11.7 (1)	0.006
High alcohol consumption (≥24 g per day)	4.1	2.3	3.3 (1)	0.07
Heavy smoker (≥10 pack years)	3.9	3.1	0.6 (1)	0.45
Physical activity limitations	9.0	31.0	167 (1)	<0.001
Comorbidity	45.7	59.9	26.6 (1)	<0.001
Cognitive impairment (MMSE <26)	6.5	9.2	4.0 (1)	0.05
Depressive symptoms (CES-D ≥16)	28.5	38.7	16.5 (1)	<0.001
*Centre*			5.6 (2)	0.06
Bordeaux	22.9	25.8		
Dijon	53.2	55.9		
Montpellier	23.9	18.3		

**Table 3:** Cause of death according to the use of hormone treatment at baseline.

Hormone Therapy	All-cause	Cancer	Cardiovascular disease	Respiratory, infectious disease	Cachexia, diseases of the digestive tract	Other causes	Ill-defined
	% (n)	%	%	%	%	%	
Never	8.0 (284)	32.8	24.3	8.5	4.2	9.1	21.1
Past HT use	6.2 (52)	44.2	17.3	7.7	5.8	5.8	19.2
Current HT use	2.2 (16)	75.0	6.3	12.5	0	0	6.2
p-value^a^	<0.001	0.32	<0.001	0.30	0.41	0.04	0.005

a Unadjusted chi-squared analysis for the difference in the frequency of deaths according to HT use.

### Association between hormone treatment and mortality

Adjusted analysis using Cox proportional hazards models, showed no significant difference in the risk of all-cause mortality between never, past and current HT users (Table 4). These differences also remained non-significant after additional adjustment for age at menopause. Examining further the characteristics of current HT, no significant difference in mortality risk was found based on the duration or type of treatment used or between women who started treatment close to the menopause versus those who initiated HT more than five years later. In terms of cancer-related death, neither current nor past HT users had a significantly modified risk of death compared to never users (past HT: multi-adjusted HR = 1.30, 95% CI: 0.82–2.07; current HT: multi-adjusted HR = 1.13, 95% CI: 0.59–2.15). No other cause-specific death could be investigated as the numbers were too small. In particular, the lower frequency of cardiovascular deaths among current HT users that was observed in unadjusted analysis could not be examined further. When all non-cancer related deaths were examined together however, current HT use versus never use was associated with a significantly reduced risk of mortality (multi-adjusted HR = 0.30, 95% CI: 0.11–0.83, p = 0.019). Past use of HT was not associated with a significantly modified risk of non-cancer related deaths (multi-adjusted HR = 0.89, 95% CI: 0.60–1.32).

**Table 4:** Cox proportional hazard models for the risk of all-cause deaths according to the use of hormone treatment at baseline.

	Women	Deaths	Follow-up,	Hazard Ratio (95% CI), p	
Hormone treatment	N	N	Person-Years	Age-adjusted	Multivariate adjusted^a^
Never	3576	284	17,568	1 (Referent)	1 (Referent)
Past	845	52	4223	1.04 (0.77–1.41), 0.78	1.04 (0.72–1.41), 0.79
Current	714	16	3645	0.62 (0.37–1.05), 0.07	0.66 (0.39–1.12), 0.12
***Characteristics of current users*** b					
≤10 years current use	274	5	1414	0.36 (0.11–1.13), 0.08	0.37 (0.12–1.16), 0.09
>10 years current use	389	10	1970	0.74 (0.42–1.33), 0.31	0.81 (0.45–1.46), 0.49
initiated ≤5 yrs after menopause	385	7	1961	0.57 (0.26–1.22), 0.15	0.62 (0.29–1.34), 0.23
initiated >5 yrs after menopause	275	8	1418	0.75 (0.37–1.54), 0.44	0.77 (0.38–1.58), 0.48
unopposed estradiol HT^c^	127	5	656	0.95 (0.39–2.32), 0.91	0.94 (0.39–2.30), 0.90
oral estradiol+progestagen HT	121	3	609	0.74 (0.23–2.33), 0.60	0.84 (0.26–2.65), 0.76
transdermal estradiol+progestagen HT^d^	439	7	2244	0.45 (0.21–0.96), 0.04	0.48 (0.22–1.04), 0.06

a Adjusted for age, education, recruitment centre, living situation, incapacities, comorbidity, depressive symptoms and cognitive impairment.

b With reference to never users.

c 113 (89%) used transdermal estradiol treatment and 14 (11%) oral estradiol.

d The 27 women who currently used other types of HT were not included in this analysis;

### Estrogen receptor polymorphism interactions

The estrogen receptor genotype frequencies for *rs2234693* were *TT* = 1351, *CT* = 2247, *CC* = 865; for *rs9340799 AA* = 1870, *GA* = 2082, *GG* = 511; for *rs1271572 GG* = 1434, TG = 2262, *TT* = 767; for *rs4986938 GG* = 1623, *GA* = 2182, *AA* = 658; and for *rs1256049 GG* = 4097, *GA* = 361, *AA* = 5. These frequencies were not significantly different from those predicted by the Hardy-Weinberg equilibrium, except in the case of the *rs1271572* (χ^2^_1_ = 6.1, p = 0.014). The *ESR1* SNPs were in strong linkage disequilibrium (|D′| = 0.98), as were the three *ESR2* SNPs (|D′|>0.90 for all pairwise comparisons). To maximise the power of the analyses when examining the potential modifying effects of these polymorphisms on the association between current HT and mortality, homozygotes for the variant allele (the smallest group in each case) were combined with the heterozygotes. Statistically significant interactions at the 5% significance level were found between current HT and three of the five SNPs on all-cause and cancer-related mortality (Table 5). One of these interactions would remain significant even if a Bonferroni correction for multiple comparisons was applied, lowering the significance threshold to 0.005 (five SNPs and two outcomes). Such a correction would, however, be overly conservative given that these tests for a HT×SNP interaction were not independent (i.e. the SNPs are in strong linkage disequilibrium and cancer-related deaths are included within all-cause deaths). The results of the analysis stratified by genotype are given in Table 5, to determine the separate associations between current HT and mortality risk. Women using HT with a C allele of *ESR1 rs2234693* had a 60% decreased risk of all-cause mortality compared to non-current users and there was a similar trend for women with the G allele of *ESR1 rs9340799*. Women with the T allele for ESR2 rs1271572 also had an almost 60% reduced risk of dying with current HT. In contrast to the findings for all-cause mortality however, current HT was found to greatly increase the risk of cancer-specific mortality for women homozygous *TT* or *AA* for *ESR1 rs2234693* and *rs9340799*, respectively, and for those with the *GG* genotype of *ESR2 rs1271572*.

**Table 5:** Cox proportional hazard models^a^ for the risk of dying associated with the use of current hormone treatment (versus non-current use) at baseline, stratified by estrogen receptor genotype.

	Multivariate-adjusted^b^ risk of mortality associated with current HT					
Genotype	All-cause				Cancer-related	
	Deaths	*HR (95% CI)*	*p*	Deaths	*HR (95% CI)*	*p*
***ESR1 rs2234693***						
*Interaction term*			0.006			0.004
*TT* (n = 1351)	85	1.65 (0.77–3.55)	0.20	32	3.18 (1.23–8.20)	0.017
*CC/CT* (n = 3112)	196	0.42 (0.18–0.97)	0.042	70	0.38 (0.12–1.27)	0.12
***ESR1 rs9340799***						
*Interaction term*			0.030			0.006
*AA* (n = 1870)	115	1.39 (0.68–2.82)	0.37	41	2.43 (1.04–5.70)	0.040
*GG/GA* (n = 2593)	166	0.42 (0.17–1.04)	0.059	61	0.31 (0.07–1.32)	0.31
***ESR2 rs1271572***						
*Interaction term*			0.060			0.019
*GG* (n = 1434)	93	1.59 (0.76–3.35)	0.22	37	2.90 (1.20–7.03)	0.018
*TT/TG* (n = 3029)	188	0.43 (0.19–0.99)	0.049	65	0.40 (0.12–1.32)	0.13

a This analysis was carried out on a sub-population of 4463 women for whom genotyping data was available.

b Adjusted for age, education, living status, recruitment centre, incapacities, comorbidity, depressive symptoms and cognitive impairment.

## Discussion

We have investigated the association between HT and mortality, focusing on characteristics of treatment and the potentially modifying effect of estrogen receptor polymorphisms. While we found no significant association between HT and all-cause or cancer-related mortality, regardless of the type of treatment, duration of use or age at first initiation, there was some evidence that HT may reduce the risk of non-cancer related deaths. Furthermore, our research suggests that the association between HT and mortality may vary depending on a woman's genetic profile. Further confirmation of this novel finding is required in another independent population based study.

### Comparison with other studies

The results of RCTs support the findings of our study in that they report no effect of HT on overall mortality [12], [13], [14], [15], [16], [41] or deaths related to all types of cancers [12], [14], [15], [16]. By contrast, the majority of observational studies have found that HT is associated with a decreased overall mortality risk [5], [6], [7], [8], [11] with mixed findings for cancer deaths [7], [8], [11]. These differences may be partly due to the healthy-users bias of women in observational studies who selectively take HT and are known to be more highly educated and with better overall health. Current HT users appeared less likely to die (all-causes) in our study compared to never users, however after adjustment for a number of health and lifestyle covariates, this association was not significant. However, it cannot be excluded that our non-significant association between HT and all-cause mortality was related to the length of follow-up and/or the small number of deaths. The observational studies which have examined specific causes of death have predominately found that the reduction in mortality with HT use was due to a large decrease in cardiovascular-related deaths [5], [8], [11]. As only one woman using HT died from a cardiovascular cause in our study, we could not perform adjusted analysis on this specific outcome, however there was a much higher percentage of cardiovascular deaths among women who had never used HT, which supports previous data from observational studies [5], [6], [7], [8], [11]. This is also supported by our finding of a 70% reduced risk of non-cancer-related deaths for HT users.

We found no significant difference in mortality risk when we examined the women currently using HT according to their type or duration of treatment, or the age when they first initiated treatment. Findings from both observational studies and RCTs indicate that HT initiated around the menopause only may lower the risk of cardiovascular disease [22], but such early initiation may increase the risk of breast [42] and overall cancers [43]. Thus the benefits of early initiation in terms of some health outcomes may be balanced out by the negative effects on others.

### Genetic variability

A novel finding of this study is the significant interaction between HT and three of the five estrogen receptor polymorphisms examined, such that the association between HT and mortality varied according to women's genetic vulnerability. Women with at least one C or G allele, for the *ESR1 rs2234693* and *rs9340799* respectively, had a significantly decreased risk of dying from all causes with current HT, while those homozygous *TT* or *AA* had a significantly increased risk of cancer-related death with HT. Likewise, women currently using HT who were homozygous *GG* for *ESR2 rs1271572* had an increased risk of death due to cancer, while those with a T allele had a reduced risk of all-cause death. Although we could not examine the interaction between HT and estrogen receptor polymorphisms on cardiovascular-related mortality specifically, this was the second most frequent cause of death after cancer. Therefore, it is possible that the reduced risk of all-cause mortality with HT for these specific genotypes, results in part from a reduction in cardiovascular-related deaths.

The biological actions of estrogen occur in large part through binding and intracellular activation of its two receptors, *ESR1* and *ESR2* [44], which can then regulate the expression of hundred of genes. Polymorphisms in the ERs could therefore influence estrogen-mediated signaling by modifying the biological potency of estrogen and thus the effect of estrogen-containing HT on health outcomes. Indeed, the *rs2234693* and *rs9340799* polymorphisms appear to be functionally significant, with the C and G alleles respectively being associated with higher gene expression [34], [35], and more favourable estrogen-dependent outcomes (e.g. bone mineral density [31]). The functional consequence of the *rs1256049* has not been established and it may be in linkage disequilibrium with other unidentified polymorphisms. To our knowledge, no previous study has investigated whether genetic variants in the estrogen receptor can modify the association between HT and mortality. Some very recent studies have examined similar polymorphisms and their interactions with HT in respect to breast cancer risk, but not mortality. Among Hispanic women, those *AA* for *rs9340799* had an increased risk of breast cancer with HT compared with women with a G allele who used HT [33], which supports our findings for cancer-related mortality. A significant increased risk of breast cancer has also been reported for women with the genotype *GG* for *rs4986938* or TT for *rs1271572* who had ever used estrogen monotherapy [23]. Such treatment was used by only a small proportion of women in our analysis, which may explain the lack of association between these genotypes and cancer-deaths with HT. There is other evidence in the literature that estrogen receptor polymorphisms can modulate the effect of HT on other health outcomes, leading further support to our findings of an effect on mortality risk. For example *rs2234693* and *rs9340799* influence the beneficial effects of HT on bone mineral density [45] and women with the *CC* genotype of *rs2234693* showed a greater increase in high-density lipoprotein cholesterol response with HT compared to other women [24]. *ESR2 rs4986938* has also been shown to modulate the decrease in total cholesterol with HT [25].

### Strengths and limitations of the study

Limitations to this study include the 6-year follow-up and small number of deaths which was insufficient to examine in detail specific causes of mortality such as cardiovascular-related deaths and specific types of cancer which are likely to be differential associated with HT (e.g. breast, colorectal). Bias could be introduced due to the exclusion of participants with missing data (7%), including a higher proportion of women who died during follow-up, thus reducing the power of the study. There is also the possibility of population stratification which we could not control for because French law prohibits the collection of data related to ethnicity. Genotype frequencies for participants who died as well as those alive at the end of follow-up however, were similar to those observed previously in white Europeans [31], [46]. As some of the data such as past HT use, age at menopause and timing of initiation of HT in relation to the menopause were collected retrospectively, there may be inaccuracies with this data, possibly explaining the lack of significant associations observed in our analysis. In terms of age at menopause however, we have previously observed a high level of reproducibility between responses at baseline and at the follow-up interviews for a subset of these participants [47]. Finally, as in all observational studies, it is possible that there are other factors which were not considered in this analysis which may have confounded the associations found in this analysis. Strengths include the sample size and population-based prospective nature of the study. Menopausal women had a broad range of health states and patterns of HT use, thus better reflecting health outcomes in the broader community. Detailed information on lifetime HT use allowed specific characteristics of treatment to be examined and comprehensive information on the mortality status of the participants was available. Estrogen receptor polymorphism data enabled genetic comparisons to be made. Replication of our findings in other large population-based studies is needed.

Findings from this study indicate for the first time that polymorphisms of the *ESR1* and *ESR2* could possibly modify the association between HT and mortality and this may help explain previous research inconsistencies. Speculatively this work suggests that women with certain polymorphisms could benefit from HT in terms of health-related outcomes, while other women may actually be genetically more susceptible to the negative effects of HT. These results require confirmation in another large population-based study. If they are confirmed they could have important clinical applications, such as suggesting the clinical utility of tailoring future prescribing according to genetic vulnerability.
